# Seasonal Movement and Distribution of Fluvial Adult Bull Trout in Selected Watersheds in the Mid-Columbia River and Snake River Basins

**DOI:** 10.1371/journal.pone.0037257

**Published:** 2012-05-24

**Authors:** Steven J. Starcevich, Philip J. Howell, Steven E. Jacobs, Paul M. Sankovich

**Affiliations:** 1 Native Fish Investigations Project, Oregon Department of Fish and Wildlife, Corvallis, Oregon, United States of America; 2 Forestry and Range Sciences, United States Department of Agriculture Forest Service, La Grande, Oregon, United States of America; 3 Columbia River Fisheries Program Office, U.S. Fish and Wildlife Service, La Grande, Oregon, United States of America; Centre of Marine Sciences & University of Algarve, Portugal

## Abstract

From 1997 to 2004, we used radio telemetry to investigate movement and distribution patterns of 206 adult fluvial bull trout (mean, 449 mm FL) from watersheds representing a wide range of habitat conditions in northeastern Oregon and southwestern Washington, a region for which there was little previous information about this species. Migrations between spawning and wintering locations were longest for fish from the Imnaha River (median, 89 km) and three Grande Ronde River tributaries, the Wenaha (56 km) and Lostine (41 km) rivers and Lookingglass Creek (47 km). Shorter migrations were observed in the John Day (8 km), Walla Walla (20 km) and Umatilla river (22 km) systems, where relatively extensive human alterations of the riverscape have been reported. From November through May, fish displayed station-keeping behavior within a narrow range (basin medians, 0.5–6.2 km). Prespawning migrations began after snowmelt-driven peak discharge and coincided with declining flows. Most postspawning migrations began by late September. Migration rates of individuals ranged from 0.1 to 10.7 km/day. Adults migrated to spawning grounds in consecutive years and displayed strong fidelity to previous spawning areas and winter locations. In the Grande Ronde River basin, most fish displayed an unusual fluvial pattern: After exiting the spawning tributary and entering a main stem river, individuals moved upstream to wintering habitat, often a substantial distance (maximum, 49 km). Our work provides additional evidence of a strong migratory capacity in fluvial bull trout, but the short migrations we observed suggest adult fluvial migration may be restricted in basins with substantial anthropogenic habitat alteration. More research into bull trout ecology in large river habitats is needed to improve our understanding of how adults establish migration patterns, what factors influence adult spatial distribution in winter, and how managers can protect and enhance fluvial populations.

## Introduction

Bull trout *Salvelinus confluentus*, and chars in general, are considered glacial relicts and have evolved several life history traits advantageous for persistence during glacial expansion and for recolonization of suitable habitat during glacial retreat [1]. Among these traits are the physiological adaptation to cold water and the ability to move long distances to find necessary resources [1], [2]. As a result, bull trout spawn and rear in or near the coldest sections of the stream network, which are usually small, high-elevation, and unproductive headwater streams [3]. They often move from these areas to larger streams, lakes, or reservoirs that provide resources for improved growth and reproductive potential [1], [4]. These habitats are distributed in a complex mosaic, varying in space and time, across a naturally fragmented riverscape [5]–[7]. In this environment, bull trout have evolved diverse migratory strategies and adaptations to local habitat conditions [2]. Life history studies of bull trout have documented resident, fluvial, adfluvial, and anadromous [8] migratory strategies and more than one of these strategies may exist in a single basin [3], [9]. Fluvial [10] and adfluvial [11] migration distances over 250 km have been reported.

This diversity in migratory behavior is important to the stability and persistence of bull trout populations [3]. The diversity and extent of fluvial migrations reflect how local populations have adapted to the spatial and temporal distribution of local habitats [12] and may provide information on the extent of suitable habitat available to each population and how life history expression is affected by human management and activities that fragment the riverscape [5]. Most of the published research on adult fluvial life history has been limited to a few basins in Idaho, Montana, and western Canada and may not be representative of the diversity of local habitat conditions within the species distribution in western North America.

In order to fill this information gap, we used radio telemetry to investigate the seasonal movement and distribution patterns of adult bull trout from basins selected to represent a wide range of habitat conditions in northeastern Oregon and southeastern Washington. Our study area consisted of basins with relatively pristine habitat conditions [13] and basins that have been severely altered by humans [14]. The three objectives of this study were to 1) quantify maximum migration distance, 2) describe timing, pattern, and rate of migration, and 3) identify general spawning and winter distributions of adult bull trout in each basin. The goals of this study were to provide information on bull trout life history to regional managers and to improve our general understanding of seasonal movement and distribution patterns of this species.

## Methods

### Study Area

**Figure 1:**
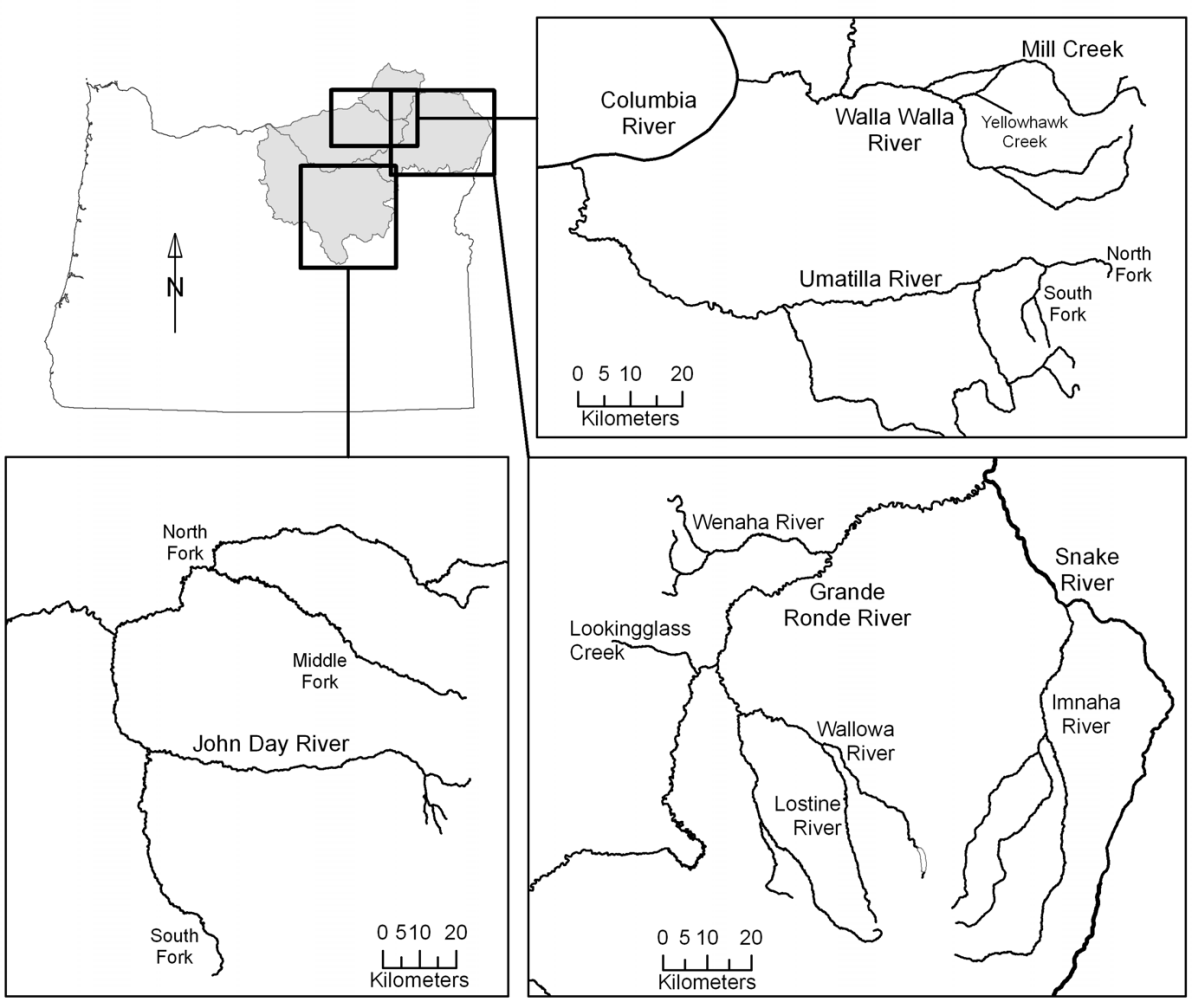
Map of the study area. Map of the study area in the Columbia and Snake river basins.

Bull trout were radio tagged in the Imnaha, Wenaha, Lostine, John Day, and Umatilla rivers, Lookingglass Creek, and in Mill Creek, tributary to the Walla Walla River (Figure 1). This region generally has a semiarid, continental climate and most precipitation falls as snow at higher elevations from November to May. In the Umatilla and Walla Walla river basins, the climate is modified by marine air from the Pacific Ocean, which brings rain in late fall and winter.

The watersheds of the Imnaha (basin area, 2,850 km^2^) and Wenaha (basin area, 760 km^2^) rivers and Lookingglass Creek (basin area, 200 km^2^) are mostly forested and within public lands designated wilderness, recreation area, or national forest (Figure 1). In the Lostine River (basin area, 240 km^2^), spawning areas are within wilderness areas but the floodplain section (river kilometer [RK] 0–10) and the adjoining Wallowa River (RK 20 upriver to Wallowa Lake) have reduced habitat quality because of residential development, stream channelization, and agricultural practices [15]. Adjacent reaches of the Grande Ronde River and Snake River have relatively low human population density and relatively high summer base flows (respective means, 21 and 504 cms). These basins contain populations of spring Chinook salmon *Oncorhynchus tshawytscha* and steelhead trout *O. mykiss*.

The spawning areas in the John Day (basin area, 20,980 km^2^) and Umatilla (basin area, 6,580 km^2^) and Walla Walla (basin area, 4,450 km^2^) river basins are mostly within forested public lands (Figure 1). The floodplain habitat has been altered by over a century of human activities [15] that have resulted in the extensive loss of riparian vegetation, channel complexity, instream large wood, and large pools [14]. There is spring Chinook salmon and steelhead trout production in the John Day River basin [15]. In the Umatilla and Walla Walla river basins, summer dewatering of large river sections was common historically and resulted in the extinction of all five wild salmon stocks [15]. In Mill Creek (basin area, 250 km^2^ at RK10), Bennington Diversion Dam (RK 18) was originally built in 1942 with no fish passage facilities and was retrofitted with a fish ladder in 1982. Through the city of Walla Walla, Mill Creek is a concrete canal with channel-spanning weirs. During summer most of the river is diverted into Yellowhawk Creek, which is a modified irrigation diversion that provides an additional connection to the Walla Walla River. Unscreened diversions on Yellowhawk Creek may obstruct upstream fish passage and entrain fish moving downstream [15].

### Fish Capture

Bull trout were caught by angling in the Imnaha River between RK 98 and 107 and Wenaha River near RK 5, 14, and 20. In Lookingglass Creek, fish were caught at a weir trap (RK 4.5) or by angling in a large pool near the trap. In the Lostine River, bull trout were caught in an upstream picket weir trap near the mouth (RK 1) and by angling upstream (between RK 17 and 39). In the John Day River basin, fish were caught by angling and in weir traps in Call Creek (RK 0.5), Deardorff Creek (RK 5), Roberts Creek (RK 1), and two locations on the mainstem (RK 437 and 450). Bull trout were captured by angling between RK 140 on the upper Umatilla River and RK 2 on the North Fork Umatilla River. In Mill Creek, bull trout were caught by angling in the pools adjacent to the municipal intake dam (RK 41) or in a trap affixed to the upstream end of its fish ladder.

### Radio Transmitters and Tagging

Radio transmitters ranged in battery life from 8 to 24 months (Lotek NTC-6-2, and Advanced Telemetry Systems models 2-357, 2-375, and 10-28) and emitted a pulsed signal at frequencies from 150 to 152 MHz. Transmitter weight did not exceed 3% of the host fish’s weight [16]. Bull trout were anesthetized prior to and during surgery with 50 mg/L tricaine methanesulfonate buffered with an equal amount of sodium bicarbonate. The transmitters were implanted into the peritoneal cavity using the previously described methods [17]. The transmitter antenna was passed through the body wall using a shielded cannula [18]. Surgery lasted less than six minutes. The fish recovered from anesthesia in a covered and aerated bath for at least 15 minutes before being released in slow, deep water near the capture site. Surgeries were not done when water temperatures exceeded 15°C.

A scientific taking permit for this research was authorized by the U.S. Fish and Wildlife Service under a cooperative agreement with the Oregon Department of Fish and Wildlife under Section 6(c)(1) of the Endangered Species Act (ESA). The terms and conditions of ESA Section 4(d) regarding authorized take and the responsible and ethical treatment of listed fish species were followed. Prior to handling fish, this research project was also reviewed by the Columbia Basin Fish and Wildlife Authority, Bonneville Power Administration, and the Independent Scientific Review Panel of the Fish & Wildlife Program of the Northwest Power and Conservation Council and its partners, NOAA Fisheries, and the Columbia River Inter-Tribal Fish Commission.

### Radio Tracking

Radio-tagged fish were tracked from the ground and air using a Lotek receiver (SRX 400). We used a handheld two-element antenna when tracking on foot and a five-element Yagi antenna when tracking by vehicle. Aerial tracking was conducted from a high-wing monoplane (Cessna 180), with two-element antennas affixed to each wing. When tracking by vehicle or foot, the transmitter location was estimated in the river by triangulating on the strongest signal [19]. Aerial tracking error was estimated by comparing empirical aerial location estimates with the corresponding known transmitter location in the study river. The aerial tracker did not know the location of the test transmitters. The interval between tracking observations differed among watersheds depending on remoteness, private land accessibility, and flight availability. Tracking occurred more frequently during the late spring through fall when fish were moving more rapidly or spawning and when more remote portions of the study drainages were more accessible.

### Description and Quantification of Movement Patterns

Two movement patterns were described: migration and winter station-keeping. Migration was defined for potamodromous salmonids by four main features: 1) sustained directional movement, 2) occurring with seasonal periodicity, 3) resulting in an alternation between at least two “well-separated” habitats, and 4) with behavioral consistency within the population [2]. Station-keeping behavior was defined as meandering and repetitive movements within a much smaller range relative to migratory movements [20].

For each fish, the following characteristics were quantified: maximum distance, timing, duration, rate, and frequency of migrations; wintering range and duration; spawning timing; and wintering and spawning site fidelity. These movement characteristics were summarized by study basin. To show general migration patterns, two locations for each bull trout were plotted on maps: 1) the farthest upstream location during the spawning period and 2) the winter modal location. The spawning period, based on previous spawning surveys in these basins, was defined as 15 August to 15 November. Migration distance was defined as the river length between the farthest upstream location during the spawning period and winter modal location. Wintering range was defined as the river reach used by a fish showing station-keeping behavior after the post-spawning period and prior to the prespawning migration. Wintering range was calculated as the distance between the farthest upstream and downstream locations during this period. Winter modal location represented where a fish was observed most often within this range.

The prespawning migration began when a fish started sustained movement away from its winter range and ended when it arrived at its estimated spawning location. Since spawning behavior was not verified by the tracker, the farthest upstream location within a known spawning area during the spawning period was used as a proxy for spawning location [21]. Spawning timing was defined as the date a fish was last observed at its estimated spawning location. The postspawning migration began when a fish was first observed departing its farthest upstream location and moving toward its wintering range. Wintering began when sustained movement away from the estimated spawning location ended and a fish began showing station-keeping behavior. Pre- and postspawning migration duration was calculated as the number of days between the winter departure date and the arrival date at the approximate spawning location between the spawning date and winter location arrival date, respectively. Migration rate (km/day) for an individual fish (*i)* is denoted by M_i_ and calculated by the following equation:
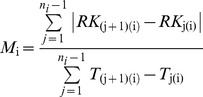
where *n_i_* is the number of observation dates for fish *i*, RK_(j)i_ represents the location (by river kilometer) of fish *i* at its *j* ^th^ observation date, and T_(j)i_ represents the *j* ^th^ observation date for fish *i*. The pre- and postspawning migration rates were calculated separately for each actively migrating fish. In calculating migration rate, to ensure an individual fish was actively migrating during a particular time period, we only used observations in which fish movement was observed on T_(j-1)(i)_ and T_(j+2)(i)_. For fish that survived through at least two spawning periods or winters, we quantified spawning and wintering site fidelity by calculating the distance between consecutive spawning sites or winter modal locations. Spawning tributary fidelity and consecutive year migration proportions were also calculated.

### Data Analysis

To determine if there were significant differences (*P*<0.05) among the basins in migration characteristics, we used one-way analysis of variance (ANOVA) and Tukey’s test for multiple comparisons. Migration distance data were log transformed to meet statistical assumptions of normality and equal variance. When the Kolmogorov-Smirnov test (with Lilliefor’s correction) indicated the data were not normally distributed, the Kruskal-Wallis test on ranks [22] was used and individual basins were compared using Dunn’s method [23] for multiple comparisons of ranked data and unequal sample sizes. Pearson product moment correlation [22] was used to evaluate the relationship between migration distance and fish length and the prespawning migration start date, between migration rates and migration distance and duration, and between Mill Creek staging behavior and arrival date in the forebay pool of the municipal intake dam, which was a unique setting and behavior in our study area.

To preserve the assumption of independence between observations in our statistical analysis, when a fish was tracked through consecutive migrations, data from only a single migration were used. Observation intervals during the spawning period may have resulted in missing some upstream movements and underestimating the farthest upstream location of an individual. To counter this potential bias in migration distances, the migration period in which the fish displayed maximum distance between spawning and wintering locations was used. In order to maximize accuracy in the migration characteristics, an individual was included if it was observed at least once every 40 days. This criterion often resulted in sample sizes smaller than those in Table 1. To ensure that tracking data used in the analysis were of transmitters in living fish, observations of an individual after its last movement were not included.

**Table 1:** Study period, sample size, fork length (FL) mean and range, and survival data of radio-tagged adult bull trout from each study watershed.

			Mean	Range	≥1^st^	≥1^st^	≥2^nd^
			FL	FL	spawn	winter	prespawn
Watershed	Year	N	(mm)	(mm)	N (%)	N (%)	N (%)
Imnaha River	2001	22	470	379–675	10 (43)	3 (15)	0 (0)
Wenaha River	1997–99	51	461	260–645	42 (82)	40 (78)	29 (57)
Lookingglass Cr.	1997–98	8	440	310–545	4 (50)	4 (50)	2 (25)
Lostine River	2001, 04	41	468	360–600	21 (51)	14 (34)	10 (22)
Mill Creek	1997–99	46	441	282–630	34 (74)	20 (43)	14 (30)
Umatilla River	2002	15	410	351–513	14 (93)	7 (47)	3 (20)
John Day River	1998–99	23	405	285–560	20 (87)	17 (74)	12 (52)

Survival categories represent the number of fish that were tracked successfully through at least the first spawning period [1^st^ spawn], into the first winter or later [1^st^ winter], and at least into a second consecutive prespawning period [2^nd^ prespawn.

## Results

### Radio Tagging and Tracking

We radio tagged 206 adult bull trout in the seven basins (Table 1). Fish fork length (FL) averaged 449 mm and ranged from 260 to 675 mm. There were no significant differences in fish fork length among the study basin (H = 9.9; *P* = 0.131; d.f. = 6). We tagged 93% of the fish between March and early September and 7% in October and November. We tracked 70% through the first spawning period without tag loss (e.g., shed or failed transmitters), 51% through spawning and at least one winter, and 34% through at least the second prespawning period (Table 1). We tracked 25% through at least two consecutive spawning periods and 20% through two consecutive winters.

The mean tracking error from comparing aerial location estimates (*N* = 15) to known transmitter locations in Mill Creek was 1.7 km (range, 0.2–3.1 km). The error associated with tracking by vehicle or on foot was not determined but presumably was much less than aerial tracking error. The time span between observations varied among the basins and between tracking periods (Table 2). The longer interval in the Wenaha River was caused in part by the relative inaccessibility of the watershed and difficulty in obtaining tracking flights.

**Table 2:** Mean interval between tracking observations of tagged fish in the spawning period (15 August –15 November) and the non-spawning period.

	Interval between tracking observations (days)			
	Spawning period		Non-spawning period	
Watershed	Mean	SE	Mean	SE
Imnaha River	8	1	23	7
Wenaha River	27	2	25	1
Lookingglass Cr.	80	12	43	14
Lostine River	12	2	21	5
Mill Creek	8	1	10	2
Umatilla River	15	1	21	3
John Day River	16	2	19	3

### Migration Distance

**Figure 2:**
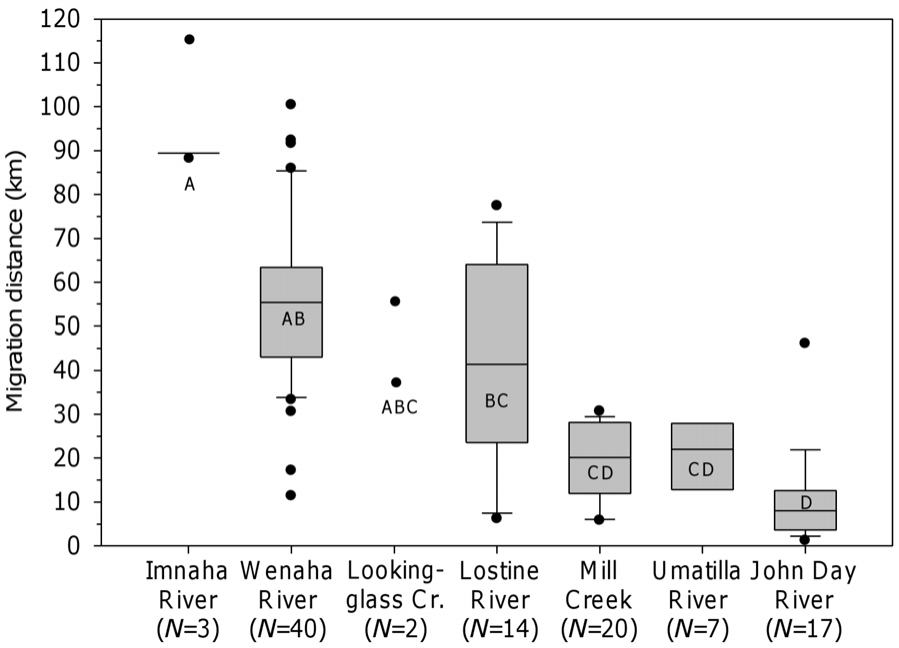
Comparison of migration distance among the study watersheds. Box plots display the median (solid line), two middle quartiles (box), 5^th^ and 95^th^ percentiles (whiskers), and outliers (black dots) for fish from each study watershed. Plots with the same letter are not significantly different (Q = 5.5–11.7; P<0.001).

Relatively long migrations were displayed by fish tagged in the Imnaha (median, 89 km; range, 89–116; *N* = 3) and Wenaha (median, 56 km; range, 11–100) rivers (Figure 2). Moderate migration distances were shown by fish tagged in Lookingglass Creek (range, 37–56; *N* = 2) and the Lostine River (median 41 km; range, 6–77). Relatively short migrations (Figure 2) were observed in fish from Mill Creek (median, 20 km; range, 6–31), the Umatilla River (median, 22 km; range, 9–33), and the John Day River basin (median, 8 km; range, 1–46). There were significant differences (F = 32.0; *P*<0.001; d.f. = 6) among the study basins in migration distance (Figure 2). There was no correlation between fish size and migration distance among fish from the Wenaha, Lostine, Umatilla, and John Day river basins (R = −0.37 to 0.31; *P*>0.20; d.f. = 5–37). There was a weak positive relationship in Mill Creek (R = 0.46; *P* = 0.05; d.f. = 17).

### General Spawning and Wintering Areas

**Figure 3:**
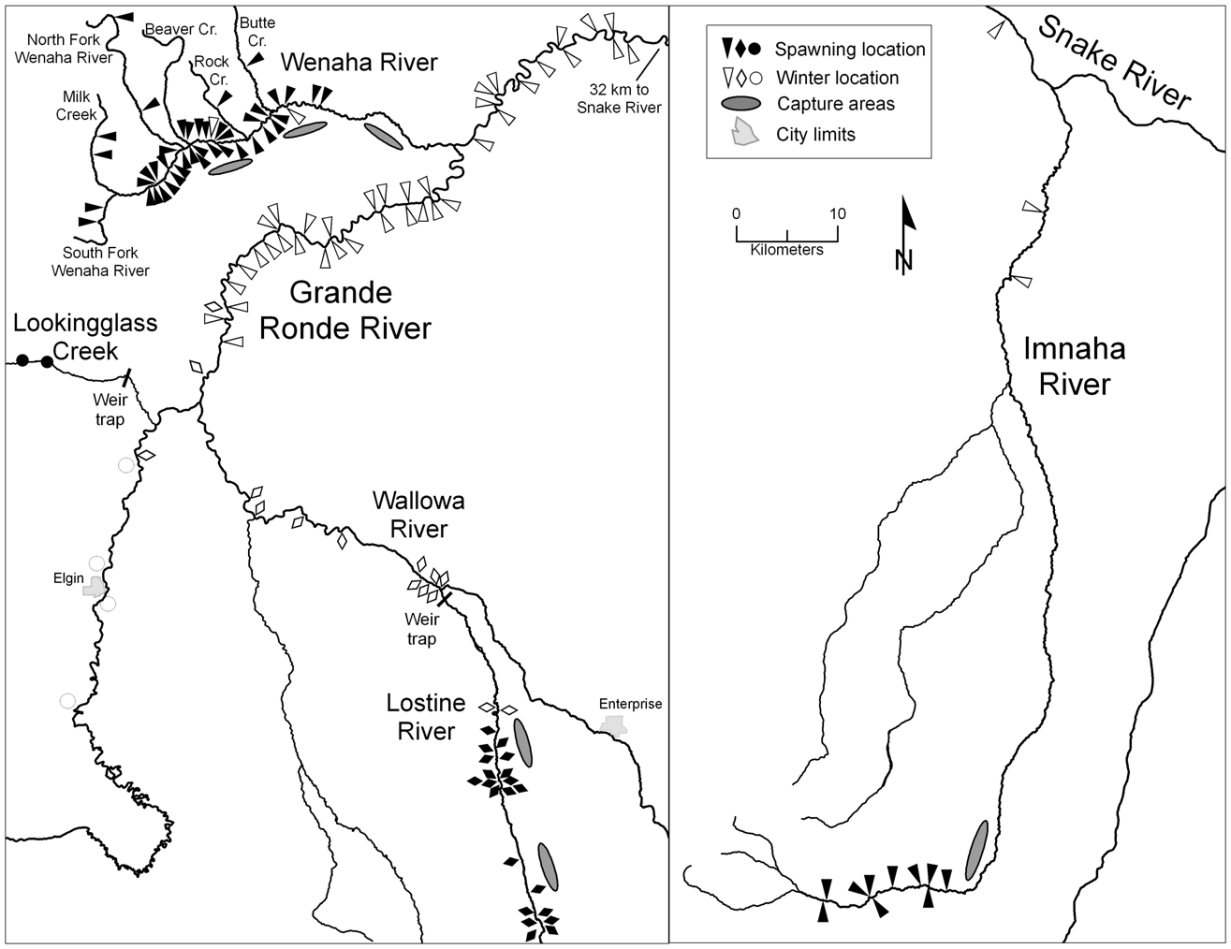
Seasonal distribution of bull trout in Grande Ronde River tributaries and the Imnaha River. Seasonal distribution of bull trout in Grande Ronde River tributaries and the Imnaha River, which include the estimated spawning locations (solid triangle) and modal winter locations (hollow).

In the Imnaha River, seven fish were tracked during the spawning period to a 15 km reach of the upper Imnaha River and three were tracked to winter locations in the lower Imnaha River and Snake River (Figure 3). The spawning distribution in Wenaha River basin started at RK 16 and continued into the upper main stem and several tributaries (Figure 3). Of 38 fish that exited the Wenaha River after the spawning period, 13 moved downstream in the Grande Ronde River to wintering areas and 25 moved upstream. These fish were distributed in winter across 86 km of the Grande Ronde River. Two fish remained all year in the upper Wenaha River and displayed short migrations (11 and 17 km), which were repeated in consecutive years. Spawning locations in the Lostine River clustered in two relatively short sections of river (5–7 km long) in the upper watershed (Figure 3). In winter, fish were distributed from the Lostine River (RK 16) to the Grande Ronde River 15 km downstream of the Wallowa River confluence, a 73 km distribution. Two fish spent all year in the Lostine River, moving less than 9 km between spawning and winter locations. In Lookingglass Creek, two fish moved into the upper watershed during the spawning period (based on one tracking observation). Postspawning fish exited Lookingglass Creek and found winter modal locations between 9 and 41 km upstream in the Grande Ronde River (Figure 3).

**Figure 4:**
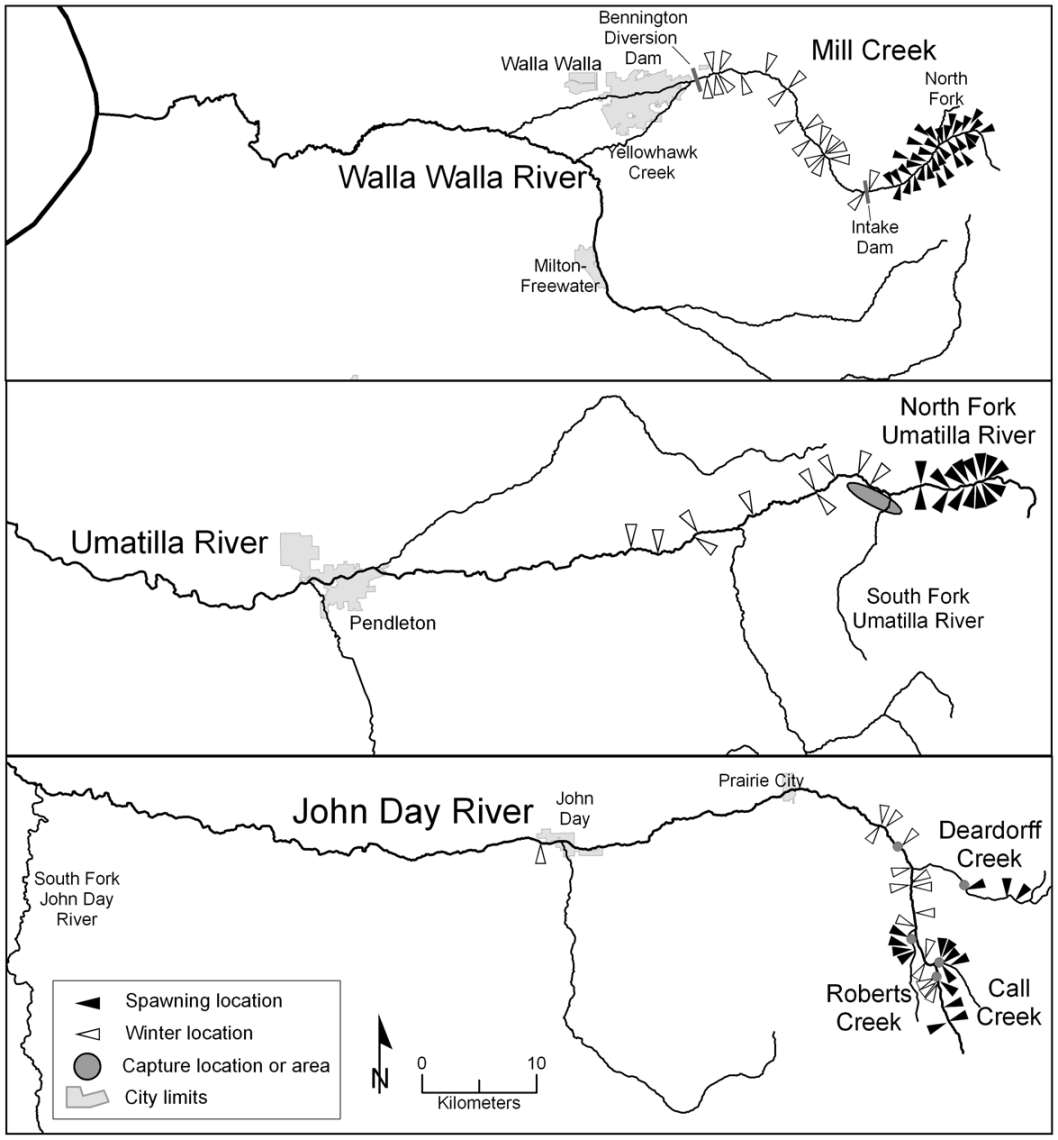
Seasonal distribution of bull trout in the Walla Walla, Umatilla, and John Day river basins. Seasonal distribution of adult bull trout in the Walla Walla (Mill Creek), Umatilla, and John Day River basins, which include the estimated spawning locations (solid triangle) and modal winter locations (hollow).

In the upper John Day River basin, fish were tracked to the upper main stem reach and its tributaries during the spawning period and into the main stem river in winter (Figure 4). The winter distribution spanned 49 km of the upper main stem; however, 94% of fish were limited to the upper 13 km of the main stem. The spawning distribution in Mill Creek spanned an 8 km reach upstream of the municipal intake dam (Figure 4). Winter locations were distributed over a 21 km reach from the intake dam to near Bennington Dam (RK 19). No fish was tracked to the reservoir created by Bennington Dam or farther downstream, although one transmitter was found on the river bank 1.4 km downstream of the dam. In the Umatilla River basin, fish were tracked during the spawning period to a 6 km reach of the North Fork Umatilla River and were distributed in the upper 24 km reach of the Umatilla River (Figure 4).

### Prespawning Migration

**Figure 5:**
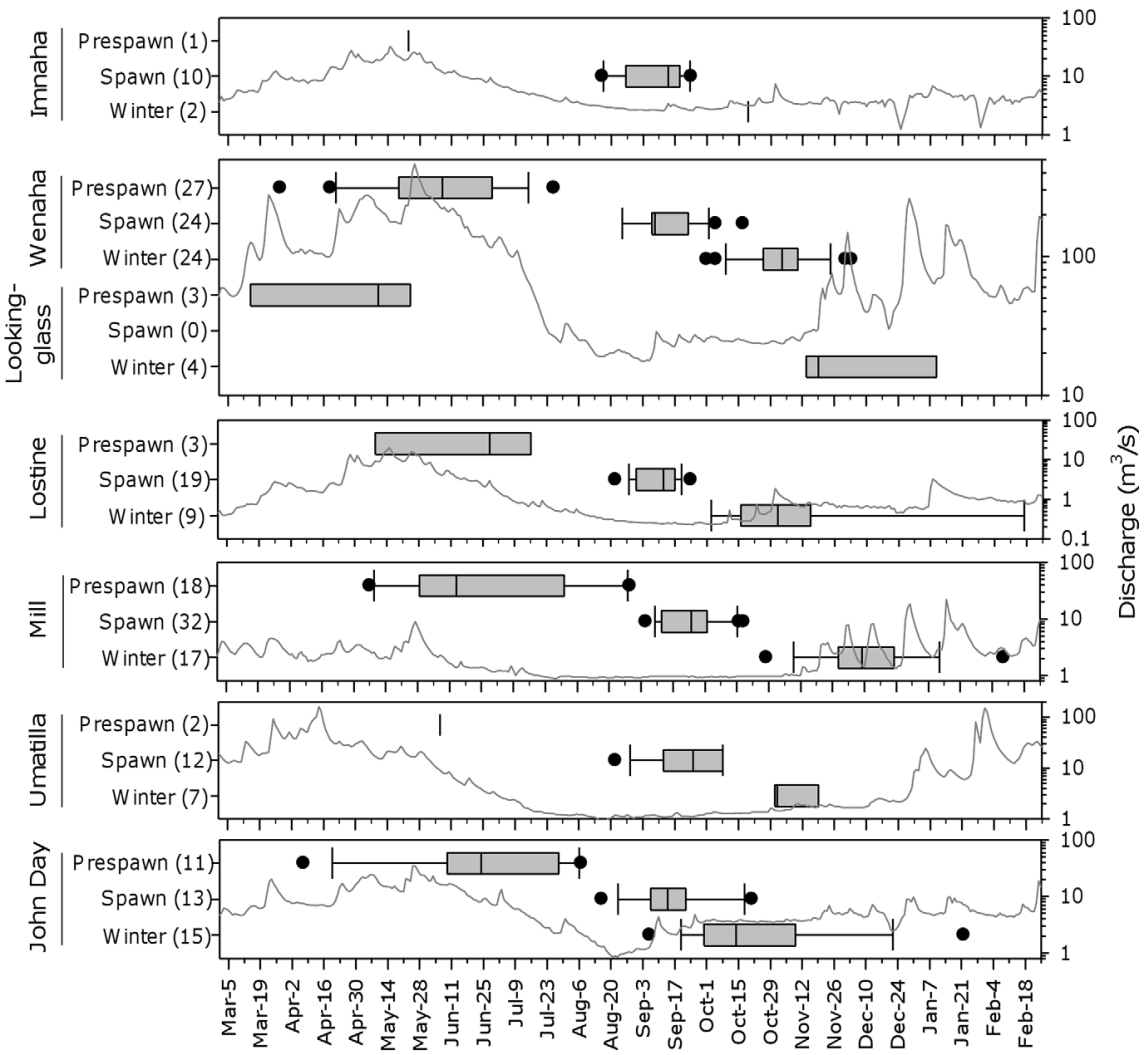
Temporal pattern of annual life history phases of fluvial bull trout in relation to discharge. Temporal pattern of annual life history phases of fluvial bull trout in relation to discharge. Boxplots, consisting of the observed start dates of the prespawning migration, spawning, and wintering for individual fish, were overlaid on mean daily discharge (gray lines; log scale) of the Imnaha River (RK 31; 2001–02), Grande Ronde River (RK 70; 1998–99), Wallowa River (RK 7; 2001–2), Mill Creek (RK; 1998–99), Umatilla River (RK 94; 2002–03), and John Day River (RK 404; 1998–99). Sample sizes are in parentheses.

Prespawning migrations began on median dates ranging from 10 May for fish from Lookingglass Creek to 28 June for Lostine River fish (Figure 5). Although the differences in start date among the basins were not significant (F = 2.36; *P* = 0.051; d.f. = 5), the median start date in Lookingglass Creek was 39 to 60 days earlier than in the other basins. Fish from the Imnaha River basin were not included in the analysis because there was only one observation. The start of the prespawning migration generally coincided with peak flows and the descending limb of the hydrograph (Figure 5). In the Wenaha and John Day river basins, there was no correlation between migration start date and distance (R = −0.09 to 0.02; *P*>0.8; d.f. = 9–23). In Mill Creek, there was a significant negative correlation between start date and migration distance (R = −0.7; *P* = 0.003; d.f. = 12), in which longer prespawning migrations were started earlier than shorter ones. We did not analyze the relationship in study basins with three or fewer sample units.

**Figure 6:**
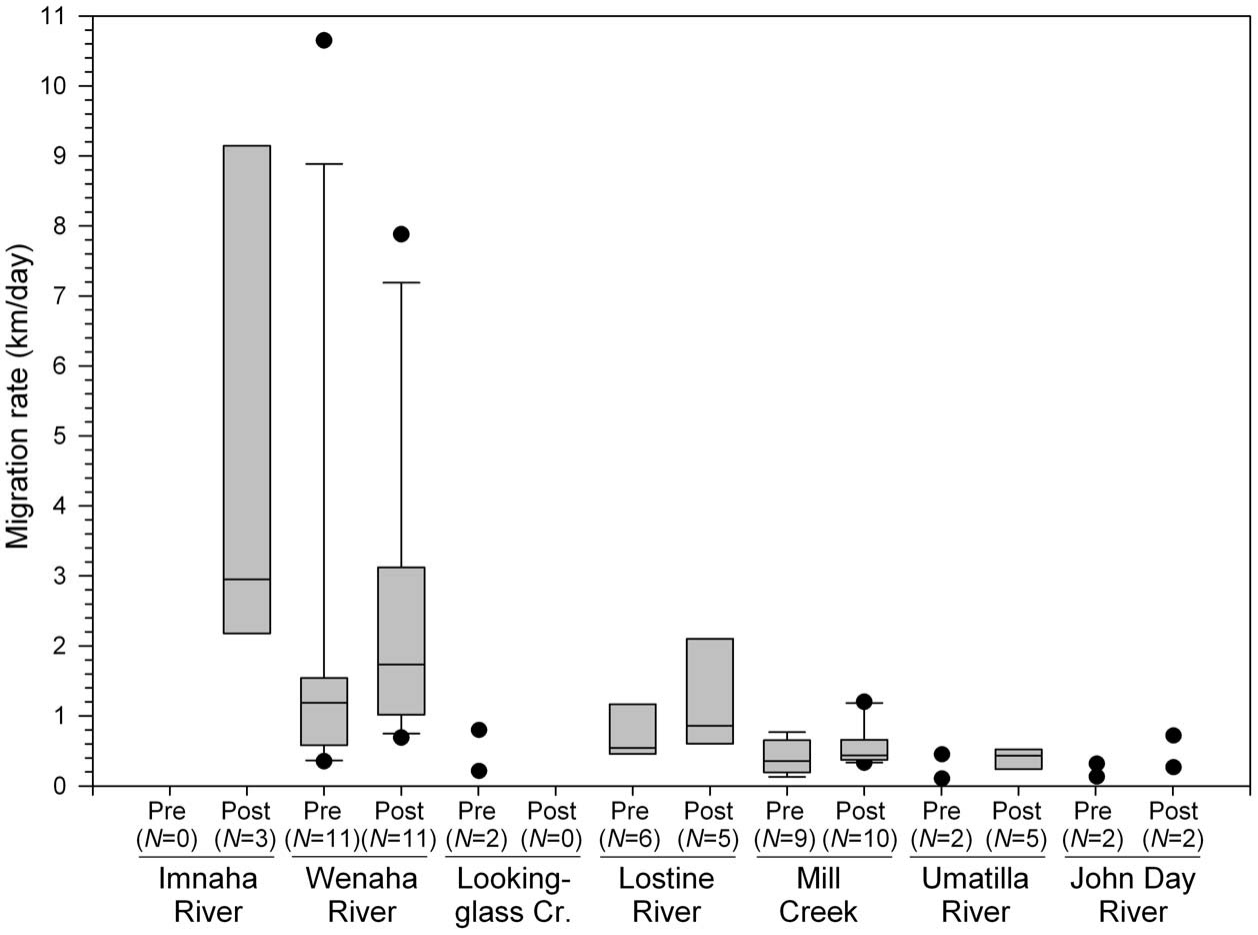
Pre- and postspawning migration rates of fluvial bull trout from the study watersheds. Pre- and postspawning migration rates of fluvial bull trout from the study watersheds.

Among the study basins, median prespawning migration rates ranged from 0.1 to 1.2 km/day (Figure 6) and duration ranged from 63 to 90 days (Table 3). Individual rates ranged from 0.1 to 10.7 km/day. Median prespawning migration rates were positively correlated with median migration distances among the basins (R = 0.87; *P* = 0.026; d.f. = 5). It was not correlated with migration duration, even with Mill Creek removed from the analysis (R = −0.60; *P* = 0.395; d.f. = 4). Mill Creek fish were not included in the analysis because they were deemed an outlier in migration duration and pattern. Most Mill Creek fish paused during their prespawning migration for an extended period (mean, 41 days) in the forebay pool created by the dam before continuing their migration. There was a significant negative linear association between the arrival date and amount of time they spent in the forebay pool, (R = −0.7, *P* = 0.002; df = 14), indicating bull trout that arrived at the pool earlier tended to remain in the pool longer.

**Table 3:** Pre- and postspawning migration duration, in days.

	Migration duration			
	Prespawning		Postspawning	
Watershed	N	Median (d)	N	Median (d)
Imnaha River	0	–	3	52
Wenaha River	19	63	19	50
Lostine River	10	68	15	57
Mill Creek	19	90	31	72
Umatilla River	5	64	7	50
John Day River	8	80	12	23

### Spawning

Median spawning timing for each watershed ranged from 8 September in the Lostine River to 25 September in Mill Creek (Figure 5) and there were significant differences among the study basins (F = 8.1; *P*<0.001; d.f. = 5). Specifically, spawning timing in Mill Creek was significantly later than in the Imnaha, Wenaha, and Lostine rivers (Q = 4.7–6.6; *P*<0.02). No other comparisons were significant (Q<3.1; *P*>0.1). Spawning timing was not determined in Lookingglass Creek as there was only single tracking observation in this basin during the spawning period.

In Mill Creek and the Wenaha, John Day, and Lostine rivers, 51 fish were tracked through 2 or more spawning periods and all migrated to known spawning areas in consecutive years. Five bull trout in the Wenaha River and four in Mill Creek were tracked to, or through, a third consecutive spawning period and all migrated between wintering and spawning areas each year. All fish tracked in consecutive years showed fidelity to the tributary basin where they spent the previous spawning period. Of these, 36 were tracked at least every 40 days during consecutive spawning periods and showed a high degree of fidelity to their estimated spawning location (Table 4).

**Table 4:** Bull trout fidelity in consecutive years to spawning and winter modal locations.

	Location fidelity (km)					
	Spawning			Winter		
Watershed	N	Median	Range	N	Median	Range
Imnaha River	0	–	–	0	–	–
Wenaha River	18	5.3	0.5–23.3	14	2.3	0.0–4.2
Lookingglass Cr.	0	–	–	1	10.6	–
Lostine River	3	0.7	0.0–1.3	1	0	–
Mill Creek	12	1.6	0.1–4.9	11	0	0.0–0.5
Umatilla River	0	–	–	1	2.7	–
John Day River	4	1.4	0.0–4.0	8	0	0.0–0.4

### Postspawning Migration

Most bull trout began their postspawning migration from September to November. Among the study basins, median postspawning migration rates ranged from 0.4 to 3.0 km/day (Figure 6) and duration ranged from 23 to 72 days (Table 3). Individual rates ranged from 0.1 to 9.1 km/day. Within a study basin, median postspawning migration rate was greater (by 22 to 160%) and duration was shorter (by 20 to 71%) than those of the median prespawning migration. However, the median differences were not great enough to be statistically significant in individuals for which pre- and postspawning migration rates were obtained (t = −1.835; *P* = 0.086; d.f. = 15). Median postspawning migration rates were positively correlated with median migration distances among the basins (R = 0.98; *P* = 0.0008; d.f. = 5). It was not correlated with migration duration, with Mill Creek removed from the analysis (R = −0.49; *P* = 0.40; d.f. = 4). During the postspawning migration in Mill Creek, bull trout arrived at the forebay pool of the municipal intake dam on the mean date of 12 October and displayed another substantial pause (mean, 45 days) in their migration.

### Wintering Behavior

**Figure 7:**
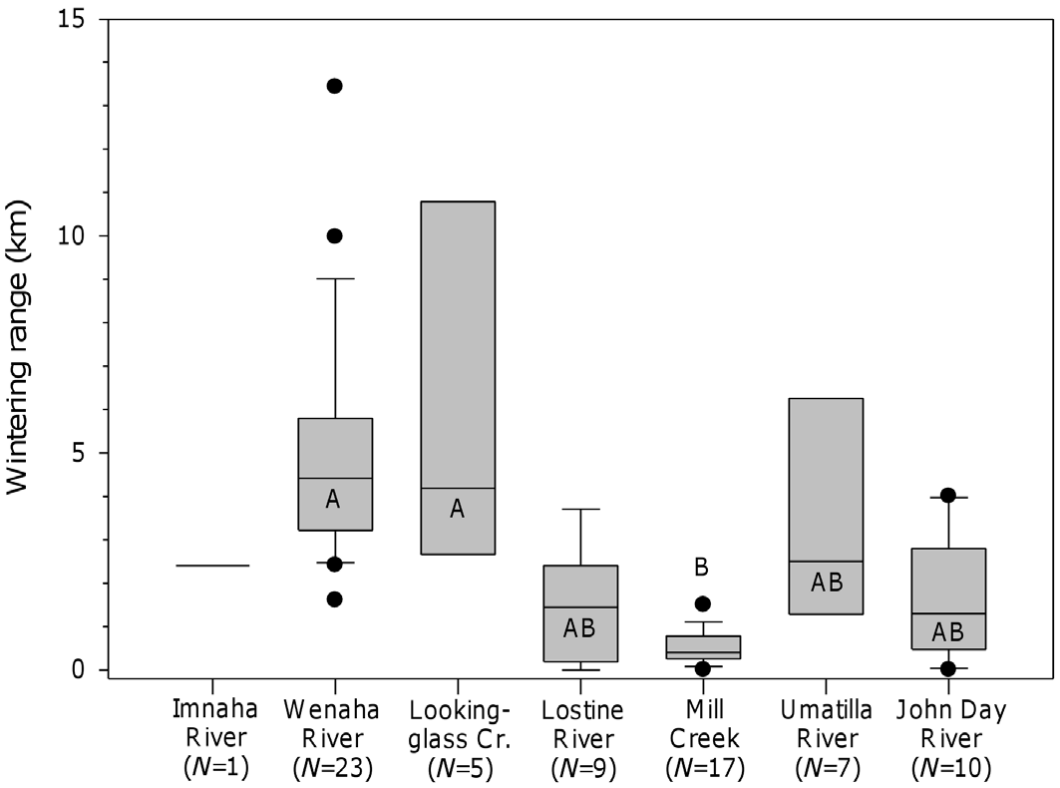
Comparison of winter range distance among the study watersheds. Comparison of winter range distance among the study watersheds. Sample sizes are in parentheses. Plots with the same letter are not significantly different (P>0.05).

Fish arrived at their winter modal location on median dates ranging from 19 October in the John Day River basin to 3 December in Mill Creek (Figure 5). Differences among the study basins were significant (H = 27.3; *P*>0.001; d.f. = 6). Mill Creek fish arrived at winter modal locations from 24 to 46 days later than other fish, but only the comparison with John Day fish was significant (Q = 4.5; *P*<0.05). Median wintering ranges varied from 0.4 km in Mill Creek to 4.1 km for Lookingglass Creek fish and 4.4 km for Wenaha River fish (Figure 7). There were significant differences among the basins in wintering range (H = 47.1; *P*<0.001; d.f. = 6). Specifically, fish from Lookingglass Creek and the Wenaha River, which wintered mainly in the Grande Ronde River, displayed significantly greater wintering range than Mill Creek fish (Q = 3.9–6.2; *P*<0.05). No other comparisons were significant. Wintering duration varied among the basins, from 172 days for Lookingglass Creek fish to 267 days in the John Day River, but there were no significant differences (Table 5). Fish tracked through at least two consecutive winters (*N* = 36) showed a high degree of winter location fidelity, returning on average to within 1.3 km (range, 0–10.6 km) of their previous modal winter location (Table 4).

**Table 5:** Wintering duration, in days, for fish from each study watershed.

Watershed	*N*	Median (d)	Min	Max
Imnaha River	1	235	–	–
Wenaha River	8	198	140	253
Lookingglass Cr.	3	172	158	179
Lostine River	5	218	133	249
Mill Creek	13	194	139	313
Umatilla River	2	206	171	241

## Discussion

Fluvial adult bull trout displayed substantial variation in migration distance and pattern among the basins in this study. Fish tagged in the Imnaha, Wenaha, and Lostine rivers and Lookingglass Creek migrated relatively long distances (medians, 41–89 km) and showed the greatest diversity of migration patterns. Migration distances were typical of large-bodied (>300 mm FL) fluvial bull trout reported elsewhere. For example, in the Salmon River basin, Idaho, migration distance of fluvial bull trout typically exceeded 68 km [24]–[26], and seasonal movements greater than 300 km have been reported [10]. In the Flathead River basin, Montana, adfluvial bull trout migrated between 88 and 250 km [11]. In the Morice River, tributary of the Skeena River, British Columbia, fluvial bull trout migrations exceeded 75 km [27]. Finally, a study in the Athabasca River, Alberta, recorded fluvial migrations over 90 km [28].

Fish tagged in Mill Creek and the John Day and Umatilla river basins displayed relatively short migrations (medians, 8–20 km) for large-bodied fluvial bull trout. In the Lostine and Wenaha rivers, two large bull trout (493 and 535 mm FL) tracked in each watershed showed relatively short migrations (6–17 km) and resided year-round near, or within, spawning areas. They represented 7% of the tagged fish in these two basins, which suggests this pattern may be relatively uncommon. This hypothesis is supported by a study that observed few adult bull trout in the Wenaha River in winter [13]. Other studies reported large-bodied fluvial bull trout with similarly short migrations, but these fish composed a small fraction of the radio-tracked population [26], [27]. Differences in fish size among the study basins were not significant and, as others previously noted [26], we found migration distance was not related to fish size. There were no known migration barriers in our study area, but there has been substantial anthropogenic habitat degradation in Mill Creek and the John Day and Umatilla rivers basins [14], [15]. In a separate analysis using data from this study, median migration distances were negatively correlated with an index for each study basin of human population density, median summer water consumption, and private land percentage [29]. However, there is little information in these study areas about the spatiotemporal distribution of resources critical to the expression of fluvial bull trout migration patterns and the effect of human activities on those resources. More information about the availability of those critical resources is necessary to understand how fluvial migration patterns are established and how these patterns are affected by anthropogenic habitat degradation. Elsewhere, there is little published research on fluvial populations of large-bodied bull trout in which such short migrations predominate. Most studies of bull trout in basins with substantial anthropogenic habitat degradation, which included at least intermittent migration barriers, concluded that the fluvial life history that was historically present in these basins had disappeared and only small-bodied (<300 mm FL) resident bull trout remained [30]–[32]. Based on the uniqueness of this pattern and the vulnerability of the fluvial life history to anthropogenic habitat alterations, these short migrations may suggest diminished habitat connectivity or patch size for these populations.

Bull trout distribution during the spawning period was similar among basins, but winter distributions varied and corresponded to differences in migration distance. Spawning areas in this study were distributed in low order tributaries and upper main stem reaches on forested, federal lands, which is typical throughout the range of the species [3]. Fish in this study generally overwintered in larger river habitats, but there was considerable variation among basins in the distribution of winter locations. Bull trout from the Wenaha and Lostine rivers found winter locations spread over long distributions (>70 km), similar to those observed among bull trout in the upper Salmon River [9], [25], [26]. In Mill Creek and the John Day and Umatilla rivers, winter locations of fluvial bull trout were distributed over a relatively short main stem reach (<25 km) adjacent to spawning areas. The narrow wintering distributions adjacent to spawning areas observed in these basins are also unique patterns not found in other populations of large-bodied fluvial bull trout. These results suggest that these migratory populations may be limited by factors downstream of their current wintering areas in larger river floodplain habitats.

Some bull trout in our study showed differences with the general directional pattern of migration of iteroparous salmonids. The typical directional pattern consists of migrating upstream to spawning grounds and downstream to winter and forage habitats where there is presumably greater potential for growth [33], [34]. Fluvial bull trout in most studies displayed this directional migration pattern [10], [25], [26], [27], [31] and many fish in this study exhibited this pattern. However, a large proportion of bull trout in this study migrated for one leg of the migration in the opposite direction of the typical migration. For example, during the first leg of the postspawning migration, almost all bull trout migrated downstream out of the Wenaha River. During the second leg, 66% (*N* = 25) migrated to upstream wintering locations in the Grande Ronde River as distant as 49 km upstream of its confluence with the Wenaha River. For fish that were tracked for multiple years, these patterns were repeated. This atypical migration pattern generally has been associated with the allacustrine life history. One study in the Pend Oreille River basin, Idaho, tracked seven radio tagged bull trout with similarly long and atypical, but mostly allacustrine, migratory patterns [34]. These fish spent the spawning period in East River and displayed postspawning migrations that involved exiting East River, moving down the Priest River to the Pend Oreille River and then upstream to winter in or near Lake Pend Oreille (38,000 ha). The lack of downstream migration in the Pend Oreille River may be the consequence of a dam that blocks upstream fish passage a short distance downstream from the Priest River junction. Other examples of this type of pattern also are associated with allacustrine forms that made either short migrations [25], [35], or longer ones [9], from spawning areas to winter and forage in upstream lakes. The existence of this atypical pattern mainly in the fluvial population from the Wenaha River basin suggests that there may be some factors limiting survival in the Grande Ronde River downstream of the Wenaha River confluence and boosting survival and reproductive success of individuals wintering in the river reach upstream. Little is known about the spatiotemporal distribution of resources in the Grande Ronde River basin and bull trout ecology in general in larger river habitats [36] so the specific factors influencing the winter distribution of this population are unknown. This atypical pattern in a fluvial context expands our understanding of the ranging ability of fluvial bull trout and how critical resources and habitats can be distributed in large river habitats, and our view of what may have been historically occupied habitats [34].

Fish in this study showed complete consecutive-year migration to known spawning areas, which is unusual relative to other fluvial populations. In the EFSF Salmon River [25] and the Blackfoot River [21], less than 33% of radio-tracked bull trout migrated to spawning areas in consecutive years. In the Morice River basin, British Columbia, 14 bull trout were tracked in consecutive years, only 3 returned to known spawning grounds, and the others migrated again long distances upstream to feed on displaced eggs behind pink salmon *O. gorbuscha* redds downstream of known spawning grounds [27]. Consecutive-year spawning reported for adfluvial populations has been similarly mixed [11], [35], [37]–[39]. Since we did not determine if individuals actually spawned, it is unknown if these adults were migrating for other reasons, such as foraging opportunities [27] or thermoregulation [21]. At least in Mill Creek, recapture and maturity data from a related study strongly suggested that bull trout were spawning in consecutive years [40]. Most of the variation among populations is likely due to variation in the productivity of particular basins and the time required for individual fish to gain the energy reserves needed for gamete production, migration, and spawning [39]. Some of the variation may be due to radio telemetry or tag recapture studies underestimating the frequency of repeat spawning by including tracking data from ejected tags or not accounting for tag loss. Such studies should specify the criteria used to ensure that data included in the study were of transmitters in living fish.

Bull trout in this study showed strong fidelity to spawning and wintering locations. All 51 fish we tracked for two to four consecutive years showed total fidelity to their previous spawning tributary and a high degree of fidelity to their previous spawning location, which is typical of other fluvial populations [21], [25], [27]. Pronounced genetic differentiation among bull trout populations [41], [42] provides further evidence that bull trout home to their natal area with high precision. Fish also showed strong fidelity to wintering locations in consecutive years and displayed station-keeping behavior in winter, which was similar to other fluvial populations. For instance, 74% of the 39 radio-tagged bull trout in the Morice River basin returned in winter to within 1 km of their previous winter location [27] and 86% of the 22 bull trout tracked in the Blackfoot River returned to within 20 m of their prior winter location [21]. Relatively short winter ranges were observed in our study as well as in other studies of fluvial populations [21], [25]. This fidelity to wintering and spawning locations suggests adult bull trout generally maintain consistent migratory patterns, with very little ranging in search of better habitats, which suggests that migration and distribution patterns of adults likely are established prior to adulthood. To gain a better understanding of how these patterns are established requires more research into the ranging behavior of juveniles and the spatial and temporal distribution of critical habitat patches affecting their growth and survival in larger river habitats [7].

Temporal patterns of migrations were similar among the study basins and other fluvial populations. The median start date of the prespawning migration occurred in May through June and did not differ significantly among the basins in our study. The initiation of migration generally coincided with the descending limb of the hydrograph, which has been noted previously [21]. There was no relationship between prespawning migration distance and start date in the Wenaha and John Day river basins. But a weak inverse relationship was found in fish from Mill Creek, which was similar to the pattern reported for bull trout in the Morice River basin [27], suggesting that the greater the distance between wintering and spawning locations, the earlier a fish began its migration. However, in both analyses less than 50% of the variation was explained in the linear relationship. Prespawning migration start date analysis was hampered by relatively small sample sizes in this study. Most fish in this study spawned in September and had begun postspawning migrations by the end of September, which is similar to previous studies of fluvial bull trout populations in Idaho and Montana [9], [21], [25], [27], [43]. We found that start dates for spawning and postspawning migration in the Imnaha and Lostine rivers, where spawning areas were found at elevations between 1200 and 1600 m in the Wallowa Mountains, were significantly earlier than in Mill Creek, where spawning occurred around 800 m elevation in the Blue Mountains. Although our study was not designed to account for the causes of variation in migration timing among the basins, differences in stream temperatures associated with climate and spawning elevation may account for the timing differences we observed.

Migration rates were similar to those previously reported for fluvial bull trout [21]. Among the study basins, migration rates were positively correlated to migration distance in both pre- and postspawning migrations, but they were not related to migration duration. This suggests that as migration distance increased, fish migrated at a higher rate. Although bull trout tended to migrate during the postspawning period at a higher rate and in less time than during the prespawning period, migration rates were calculated from observations that were from 3 to 22 days apart and fish behavior was rarely determined during tracking. Therefore, migration rate was summarized over coarse and varying time periods and some observations during the prespawning period likely were of spawning fish, which may have decreased the migration rate and increased migration duration during this period. The pre- and postspawning migratory behavior of Mill Creek bull trout was unusual relative to other case studies of migratory bull trout. During these migrations, bull trout paused for a substantial period in the large forebay pool formed by the municipal intake dam, which led to relatively longer postspawning migration duration for Mill Creek fish. Prespawning staging behavior by bull trout has been observed at the mouths of spawning streams [11], but not for the duration we observed. The forebay pool is at least four times larger than any pool in upper Mill Creek and may simply provide superior habitat for potential prespawning behavior such as mate selection, which may occur during staging [11], and postspawning recovery.

The migratory patterns observed in the Imnaha River and Grande Ronde River tributaries provide additional evidence of long distance migration among bull trout between spawning and winter habitats and highlights for regional managers the importance of habitat connectivity from headwater spawning areas to larger rivers. The pattern of short migrations and narrow wintering distributions adjacent to spawning areas that was observed in some study basins is of management concern because it suggests a potential increase in isolation and a potential reduction in habitat patch size, the consequences of which are diminished population abundance and metapopulation dynamics and an increased risk of long-term extinction [44]. Larger river habitats, especially in floodplains, are the most likely to be altered by human activities [45] and can provide important habitat for fluvial bull trout. However, there is currently little understanding about bull trout ecology during their occupation of these large river habitats [36] and the factors that limit migratory and distribution patterns. More research into these factors will lead to a better understanding of how adult bull trout establish migration patterns, what factors restrict their migration and distribution patterns, and how managers can protect and enhance fluvial bull trout populations.
